# The effects of voice content on stress reactivity: A simulation paradigm of auditory verbal hallucinations

**DOI:** 10.1016/j.schres.2019.07.019

**Published:** 2022-05

**Authors:** David Baumeister, Emmanuelle Peters, Jens Pruessner, Oliver Howes, Paul Chadwick

**Affiliations:** a Institute of Psychiatry, Psychology & Neuroscience, King's College London, Department of Psychology, London, UK; b Department of General Internal Medicine and Psychosomatics, University Hospital Heidelberg, Germany; c South London and Maudsley NHS Foundation Trust, Bethlem Royal Hospital, Beckenham, Kent, UK; d Department of Psychology, University of Constance, Constance, Germany; e Institute of Psychiatry, Psychology & Neuroscience, King's College London, Department of Psychosis Studies, London, UK; f Department of Psychology, University of Bath, Bath, UK

**Keywords:** Stress, Psychosis, Auditory hallucinations, HPA axis

## Abstract

**Objectives:**

Psychosis is associated with increased subjective and altered endocrine and autonomic nervous system stress-reactivity. Psychosis patients often experience auditory verbal hallucinations, with negative voice content being particularly associated with distress. The present study developed a voice-simulation paradigm and investigated the effect of simulated voices with neutral and negative content on psychophysiological stress-reactivity, and the effect of mindful voice-appraisals on stress-reactivity.

**Method:**

Eighty-four healthy participants completed the Montreal Imaging Stress Task with simultaneous presentation of one of three randomly allocated auditory stimuli conditions: negative voices, neutral voices or non-voice ambient sounds. Subjective stress-levels and mindful voice-appraisals were assessed using questionnaire measures, and cortisol and α-amylase levels were measured using saliva samples.

**Results:**

ANOVA revealed a significant effect of condition on subjective stress-levels (p = .002), but not cortisol (p = .63) or α-amylase (p = .73). Post-hoc analyses showed that negative voices increased subjective stress-levels relative to neutral voices (p = .002) and ambient sounds (p = .01), which did not differ from each other (p = .41). Mindful voice-appraisals were associated with less distress across conditions (p = .003), although negative voices were also associated with less mindful appraisals (p < .001).

**Conclusions:**

Negative voice content, rather than voices or auditory stimuli per se, is linked to greater subjective but not physiological stress-reactivity. Mindful appraisals may partially moderate this effect. These findings highlight the importance of voice content for the impact of voice-hearing, and highlight the potential value of mindfulness training to treat voice distress in psychosis.

## 1 Introduction

Psychotic disorders affect about 2% of the population and are characterised by a number of clinical features including delusions and auditory verbal hallucinations (AVHs) (Howes and Murray, 2014). People with psychotic disorders show increased subjective reactivity to stress (Myin-Germeys and van Os, 2007), altered function of the hypothalamic-pituitary-adrenal (HPA) axis (Bradley and Dinan, 2010; Pruessner et al., 2017), as typically measured via the glucocorticoid hormone cortisol, as well as the autonomous nervous system (ANS) (Montaquila et al., 2015), measured through heart-rate, skin conductance, or the salivary marker α-amylase (Ieda et al., 2014; Inagaki et al., 2010). Moreover, stress exposure can exacerbate the intensity of psychotic experiences in patients (Myin-Germeys and van Os, 2007; Reininghaus et al., 2016), and is associated with significantly greater striatal dopamine signalling in psychosis patients and at-risk populations compared to healthy controls (Mizrahi et al., 2012). This suggests that stress exposure could be a risk factor for the onset or exacerbation of psychosis. However, it has also been proposed that the elevated stress response seen in people with psychotic disorders is a consequence of psychosis (Howes and Murray, 2014).

The negative content of psychotic experiences may be particularly important in elevating the stress response. AVHs occur in healthy members of the general public as well as people with psychotic disorders (clinical voice-hearers) and do not necessarily cause distress or imply need for care in the healthy voice hearers (HVHs) (Baumeister et al., 2017; Johns et al., 2014). A consistent finding is that clinical voice-hearers (CVHs) experience predominantly negative voice content, whereas HVHs typically report neutral or positive voice content (Baumeister et al., 2017; Johns et al., 2014). Several studies have further reported that negative voice content is associated with greater service use and need for care in CVHs (Beavan and Read, 2010; Daalman et al., 2011; Johns et al., 2014). Within clinical populations, greater negative voice content is associated with more voice-distress, depression and low self-esteem (Smith et al., 2006). It has been argued that negative voice content may be crucial in driving the pathological impact of voice-hearing in CVHs relative to HVHs (de Leede-Smith and Barkus, 2013). Finally, there is evidence that psychophysiological stress-reactivity differs in CVHs and HVH (Baumeister et al., under review). Thus, negative voice content may contribute to the distress experienced by CVHs, and exacerbate the impact of stressful situations.

Cognitive models of psychosis have posited that the appraisal of anomalous experiences, and not simply the content, shapes their impact (Chadwick and Birchwood, 1994; Garety et al., 2001). Mindful appraisals of AVHs, that is, experiencing them with present-moment awareness, a non-judgmental attitude and ultimately acceptance, is negatively associated with voice-distress and mood problems (Chadwick et al., 2009, Chadwick et al., 2016; López-Navarro et al., 2015; Morris et al., 2014; Shawyer et al., 2007). Mindfulness-based intervention studies have reported reductions in voice-distress in CVHs (Chadwick et al., 2009; López-Navarro et al., 2015), and that HVHs react more mindfully to AVHs (Peters et al., 2016). Furthermore, mindfulness is associated with lower subjective and physiological stress-reactivity (Brown et al., 2012; Bullis et al., 2014; Daubenmier et al., 2014). Thus, whilst it is likely that there is a direct impact of voice content on stress reactivity, these responses may be moderated by ‘mindful’ voice-appraisals.

The evidence discussed above suggests that both the content of voices and the way an individual appraises experiences may play a role in the impact of voice hearing on the stress response. In view of this, we aimed to test the effect of simulated voices and their content on stress-reactivity in general population volunteers. To this end, we developed an AVH simulation paradigm that exposes participants to either negative or neutral voice content, a potentially valuable tool currently lacking in AVH research (freely available from the authors upon request). We hypothesised that simulated AVHs with negative voice content would increase the subjective, cortisol and α-amylase reaction to a psychosocial stress paradigm over and above the levels produced by neutral voices or a non-voice ambient control. The latter two were hypothesised not to differ. It was further hypothesised that a more mindful appraisal of voices during the paradigm would be associated with a lower subjective, cortisol and α-amylase stress response.

## 2 Methods

### 2.1 Sample

The sample consisted of 84 participants from the general population. Participants were primarily recruited through opportunity sampling. Participants were excluded if they were: under 18, not fluent in English, hearing-impaired, had previously experienced AVHS, had received secondary care for any mental health issue, or scored 10 or above on the depression subscale of the Hospital Anxiety and Depression Scale (Zigmond and Snaith, 1983). The latter two eligibility criteria were decided upon to exclude vulnerable individuals. We decided not to exclude siblings of psychosis patients. As our research is grounded in a psychosis spectrum model, it would have also necessitated us to exclude siblings of individuals with AVHs but no clinical distress. We felt that this could not be reliably ascertained or verified, as experiences of AVHs are likely to be very private experiences for many people. Ethical approval for the research study was granted by the King's College London PNM Research Ethics Sub-Committee (PNM/14/15-111).

### 2.2 Measures

#### 2.2.1 Montreal Imaging Stress Task

The Montreal Imaging Stress Task (MIST) (Dedovic et al., 2005) is a well-established paradigm designed to deliver a standardised psychosocial stress, utilised here outside the context of neuroimaging. In the initial training phase, participants were presented with mental arithmetic exercises on a laptop for a total of 10 min. The computer algorithm adapts to the individual's ability by making the task a bit harder (both in task difficulty and allotted time) than what the participant is capable of solving. As a result, the individual performs poorly without making it obvious that the task is rigged. The task relies on deception of participants, in that they are made to believe their performance is below what is expected and needed for the study. Participants are presented with standardised negative visual feedback on their performance by the program (i.e., ‘incorrect’, ‘timeout’, and a comparison of individual performance with (fake) average performance), and standardised negative verbal feedback by the experimenter (e.g., “Your performance is below of what we normally see, can you please try harder?”). The MIST has been utilised in numerous clinical and non-clinical studies and reliably increases subjective, neurological and physiological indicators of stress (Geva et al., 2014; Hernaus et al., 2015; Mizrahi et al., 2014; Voellmin et al., 2015; Zschucke et al., 2015). Participants are fully debriefed after the task is finished and no adverse effects have been reported.

#### 2.2.2 Voice-simulation

Three 10 min audio materials were developed for the present study: a) negative simulated voices (e.g., “What a waste of space”), b) neutral simulated voices (e.g., “Today is the day”), and c) non-voice neutral sounds (i.e., the sound of water running). To ensure validity of the voice simulation, an initial longlist of negative and neutral voice comments was drawn from a) online first-person reports of voice content in service user forums, b) service user literature and c) reports from clinicians with expertise in working with psychosis patients (sources listed in Supplement A). The final shortlist was established excluding comments that were performance- and task-specific, or too derogatory and/or potentially risky (e.g., commands to self-harm). Voice-tracks were identical in the number of statements, the number of first-person statements, as well as the frequency of words within the English language (assessed using Subtlex-UK (Van Heuven et al., 2014)). The tracks were designed to deliver comments for 10 min, with a total of 170 comments in each. The comments were read out by two male and two female volunteers who were instructed to maintain a neutral intonation to avoid confounding content with intonation. Time intervals between voices were matched for the negative and neutral tracks, as well as which speaker delivered the specific statement. The number of statements delivered by female and male voices was even. The non-voice control track consisted of a 10 min recording of water streams. See the Supplementary materials for a full breakdown of the voice-tracks.

#### 2.2.3 Southampton Mindfulness Questionnaire

The Southampton Mindfulness Questionnaire (SMQ) is a 16-item self-report measure that assesses mindful awareness of distressing voices (Chadwick et al., 2008). For the present study, mindfulness of voices during the experiment was assessed (e.g., “When I heard the voice in the experiment, I just listened and let it pass”). Items are scored on a 7-point Likert scale from 1 to 7, with total scores ranging from 16 to 112, and higher scores indicating greater state mindfulness. Its validity has been shown in both clinical and healthy samples (Chadwick et al., 2008). Cronbach's α in the present study indicated good reliability (0.87).

#### 2.2.4 Stress reactivity visual analogue scales

An 8-item visual analogue scale (VAS) was created to assess subjective stress-reactivity before and after the task, ranging from 0 cm to 16.5 cm, with total scores ranging from 0 to 132 and higher scores indicating greater subjective stress levels. Participants were asked how stressed, anxious, angry, relaxed (reverse coded), threatened, embarrassed, socially judged and expecting of positive vs negative consequences they were, to reflect an array of possible stress responses. A Principal Component Analysis was carried out to determine whether the scale represented a latent factor indicative of a general subjective stress reaction, using VAS scores at post-MIST. The Kaiser-Meyer-Olkin measure was used to verify sampling adequacy (KMO = 0.84) and Bartlett's test of sphericity indicated sufficiently large correlations (χ^2^ (28) = 259.8, p < .001). Only one component had an eigenvalue over Kaiser's criterion of 1, explaining 50.49% of the variance. Since no item had a coefficient below 0.5 (Field, 2014), all 8 items were included, with the lowest factor loading being 0.61. VAS sum scores were calculated for a total measure of subjective stress levels. Cronbach's α indicated good overall reliability (0.86).

#### 2.2.5 Control variables

To investigate potential confounding variables that may lead to variation in salivary biomarker data, a questionnaire was created to control for biological factors that may impact on physiological stress-function. Items assessed included age (Holochwost et al., 2017; Skoluda et al., 2017), gender (Kirschbaum et al., 1999), Body Mass Index (BMI) (Skoluda et al., 2017), native language (for voice content), current medications, current medical diagnoses, time of last meal, time of last drink, time of last cigarette (if applicable (Skoluda et al., 2017)), first day of last menstrual cycle (if applicable (Kirschbaum et al., 1999)), strenuous exercise in the preceding 72 hours (h) (Bonato et al., 2017), stress exposure in the preceding 24 h (Skoluda et al., 2017), and illicit substance use in the preceding 72 h (Seibert et al., 2014). A composite score for menstrual cycle and progesterone treatment was created using stratification by follicular and luteal phase and gender, so that women in luteal phases and men were grouped, and women in follicular phases and oral contraceptive users were grouped. This is in line with previous research demonstrating the effect of these variables on salivary cortisol reactivity to psychosocial stress (Kirschbaum et al., 1999). Participants with substance use in the preceding 72 h were excluded.

#### 2.2.6 Cortisol & α-amylase measurements

Saliva was sampled using Salivettes (Rommelsdorf, Germany). All Salivettes were frozen at −20 °C in a secure laboratory freezer within 2 h of collection. Participants were asked to gently chew the Salivettes for each collection, and were instructed not to touch samples with their hands. All samples were analysed at the StressLab (Trier, Germany) using ELISA-assays for cortisol and α-amylase, with an inter-assay variability coefficients of 5.2% and 3.0%, respectively.

### 2.3 Procedure

**Fig. 1:**
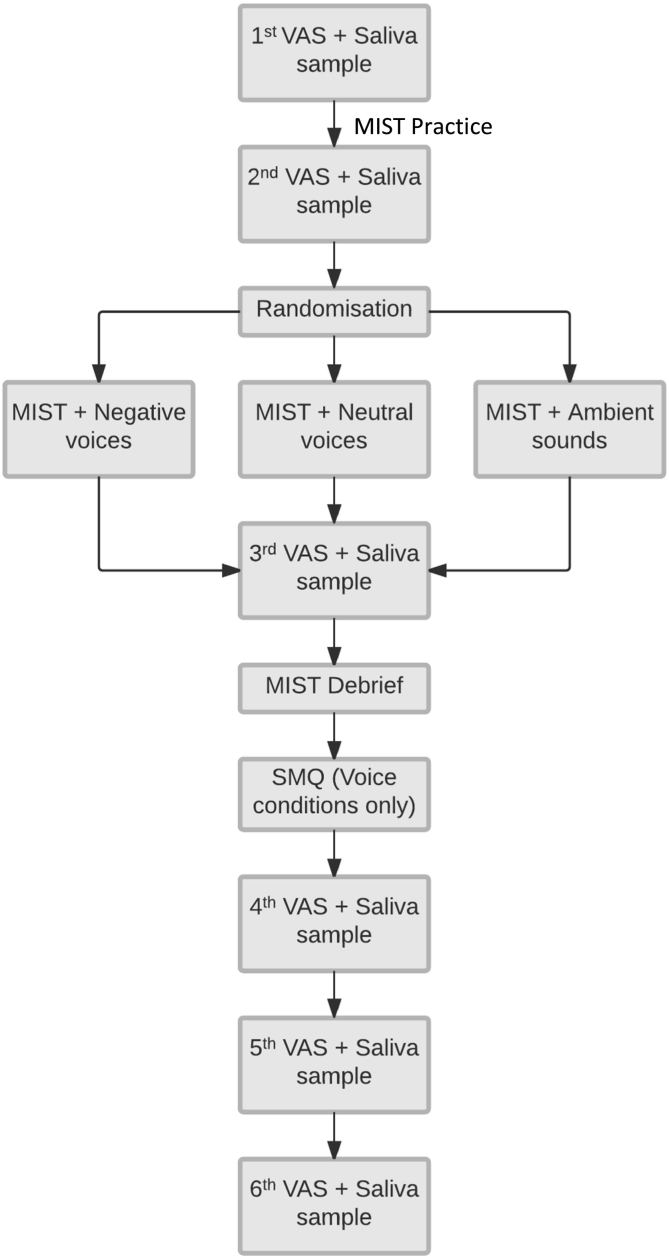
Flow diagram of experimental procedures; time between 1st and 2nd, and 2nd and 3rd VAS = 10 min, time between subsequent VAS = 15 min (MIST: Montreal Imaging Stress Scale. SMQ: Southampton Mindfulness Questionnaire; VAS: Visual Analogue Scale).

Participants were told that the aim of the study was to test the effects of auditory stimuli on cognitive abilities. Eligible participants completed an initial VAS and saliva sample, and were then invited to practice on the MIST (i.e., they completed equations without a time limit, and without the audiotrack) for 10 min. All participants were instructed to use the keyboard with their non-dominant hand to increase difficulty. During the actual trial, participants wore a headset with the headphone covering their left ear (with the experimenter sitting to their right) to present auditory stimuli, with equal volume settings for all participants. Auditory stimuli were block-randomized (www.sealedenvelope.com; blocks of 12) so that there were 28 participants in each condition, and organised by a collaborator (O.S.; acknowledgements) so that the experimenter was blinded to individual conditions. Participants received no instructions on a particular response style (e.g., mindfulness) to the auditory stimuli and were asked not to comment on the content of the auditory stimuli during their performance to avoid unblinding the experimenter. Procedures are detailed in Fig. 1.

Participants were asked to complete the second VAS and saliva sample, and then started the MIST experimental condition for 10 min whilst listening to the audio stimuli. Participants in all conditions were exposed to criticising negative social feedback by the experimenter, including questioning of their arithmetic abilities, their effort in the experiment, as well as warnings that they would be excluded if their performance did not improve. The experimenter did not respond to any queries made by the participants. Following completion of the MIST, participants were immediately asked to complete the third VAS and saliva sample. They were then fully debriefed about the true nature of the task, and participants in the two voice conditions completed the SMQ. Participants then completed the assessment of control variables, and three more VAS and saliva samples were taken in intervals of 15 min.

### 2.4 Statistical analysis

The required sample size was calculated using G*Power for a one-way ANOVA with 3 groups, assuming effect sizes of medium to large magnitude (f = 0.35) and statistical power of 0.8. Effect size was based on meta-analytic findings of differences in cortisol peak response to acute stress between schizophrenia patients and healthy controls (Ciufolini et al., 2014). This was chosen as the nearest available effect size for orientation due to the novelty of the present paradigm. Statistical analyses were carried out using SPSS for Windows (IBM Corp. Released, 2015). Normal distribution of data was checked by Q-Q plots, and Levene's test confirmed homogeneity of variance between the three conditions. To establish normal distribution of cortisol and α-amylase data, logarithmic transformations (log_n_) were undertaken for all analyses. For all cortisol and α-amylase analyses, associations of dependent variables with all potential covariates were assessed and included as covariates if significant. For cortisol, bivariate correlations revealed gender and menstrual cycle as covariates. For α-amylase, no covariates were identified. A repeated-measures mixed 6 (sampling timepoints) × 3 (condition: negative voices; neutral voices; ambient sounds) ANCOVA was carried out on the total VAS scores, cortisol and a-amylase. Lower-order repeated-measures ANOVAs using total VAS scores were used for post-hoc testing of condition-specific effects (only when a significant effect of condition, or interaction with condition, was present in the initial analysis). For tests for associations, the area under the curve with respect to the ground (AUC_g_), i.e., the total amount of biomarker secretion across all timepoints(Pruessner et al., 2003) was calculated. Bivariate correlations with VAS scores, AUCg cortisol and α-amylase were utilised for associations with SMQ. P-values below the two-tailed 0.05 threshold were accepted as statistically significant. False Discovery Rate correction for multiple testing was applied to analyses with multiple post-hoc testing, and FDR-adjusted p-values are reported where indicated.

## 3 Results

### 3.1 Descriptives

One-way ANOVAs showed effective randomization, with no significant differences in age (F(2,81) = 0.03, p = .97), gender (F(2,81) = 1.16, p = .32), BMI (F(2,81) = 1.4, p = .25) or number of native English speakers (F(2,81) = 0.96, p = .39) between conditions. One-way ANOVA also confirmed that VAS scores did not differ between conditions at pre-MIST (F(2,81) = 0.64, p = .85), and neither did cortisol (F(2,81) = 0.51, p = .51) or α-amylase (F(2,81) = 0.77, p = .47). Demographics and questionnaire scores by condition are presented in Table 1. Two participants in the negative voices condition decided to abort the MIST after 8 min but nevertheless participated in the rest of the study and were therefore included in the analyses. Two participants in the negative voices condition accidentally unblinded the experimenter but were included as their data were not indicative of outliers.

**Table 1:** Demographics and questionnaire scores by condition (mean ± SD unless specified otherwise).

	NEG (*n* = 28)	NEU (*n* = 28)	AMB (*n* = 28)
Gender (% female)	75.0%	82.1%	64.3%
Age (y)	25.9 ± 7.6	26.4 ± 8.5	26.0 ± 5.9
Native language (% English)	64.3%	71.4%	53.6%
VAS overall delta^a^	5.5 ± 1.9	3.2 ± 1.7	3.9 ± 2.5
SMQ^a^	66.2 ± 14.1	82.0 ± 16.0	–
Cortisol AUCg (log)	5.5 ± 0.6	5.6 ± 0.6	5.7 ± 0.7
α-Amylase AUCg (log)	9.3 ± 0.8	9.4 ± 0.6	9.2 ± 0.6

Note: NEG – negative condition; NEU – neutral condition; AMB – ambient condition; VAS – Visual Analogue Scale; SMQ – Southampton Mindfulness Questionnaire.

a Statistically significant difference between groups.

### 3.2 Subjective effects

**Fig. 2:**
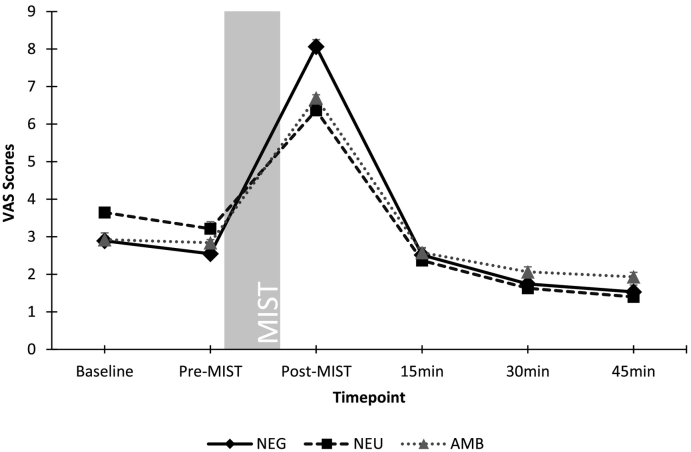
VAS scores by group by timepoint (mean ± SE).

**Fig. 3:**
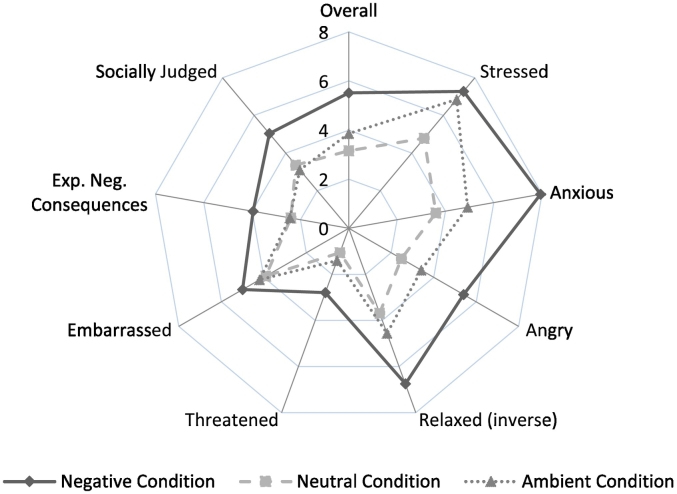
Delta scores from pre- to post-MIST for overall and individual VAS item scores by individual.

Repeated-measures ANOVA confirmed a significant effect of timepoint on VAS scores (F(3, 198) = 234.1, p < .001), and there was a significant interaction with condition (F(5,198) = 4.8, p < .001). Tests of between-conditions effects showed no significant effect of condition (F(2,81) = 0.04, p = .961). Lower-order ANOVAs were carried out including pre- to post-MIST VAS scores, to capture the dynamic of the stress response, using FDR-adjusted p-values to adjust for multiple comparisons. This showed significant condition differences in VAS change (F(2,83) = 9.7, p = .002), with significantly greater change in NEG compared to NEU (p = .002) and NEG compared to AMB (p = .01), but no differences between NEU and AMB (p = .41). VAS scores throughout the paradigm are shown in Fig. 2. Mean deltas from pre- to post-MIST for individual as well as total VAS scores are presented in Fig. 3 to illustrate change in stress levels from baseline by condition.

### 3.3 Biomarker effects

**Fig. 4:**
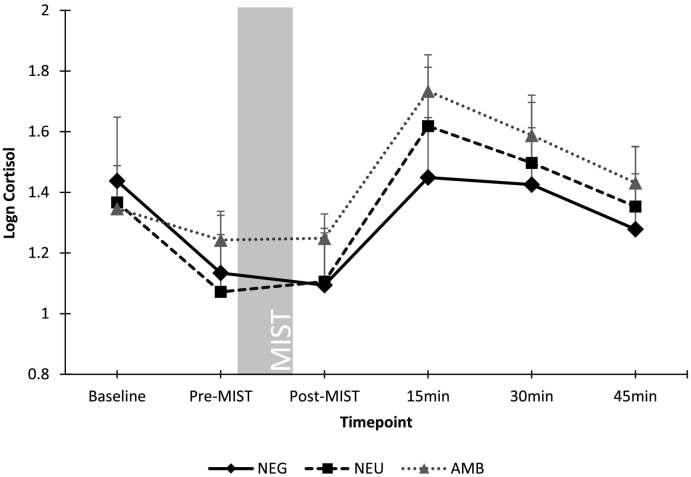
Cortisol by group by timepoint (mean ± SE).

**Fig. 5:**
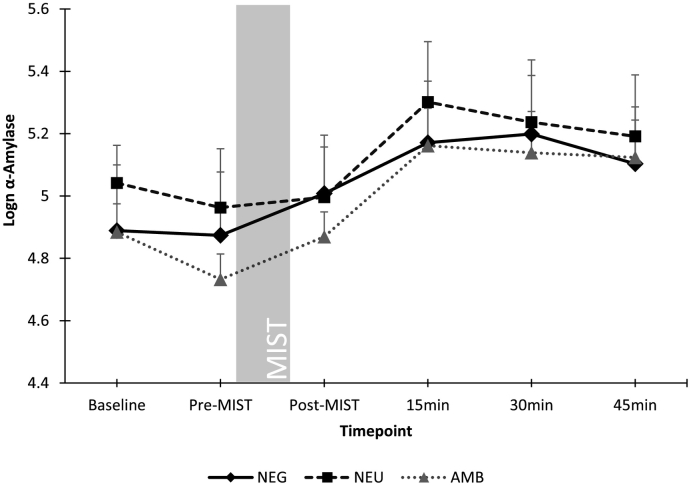
α-Amylase by group by timepoint (mean ± SE).

Repeated-measures ANCOVA controlling for gender and menstrual phase confirmed a significant effect of timepoint on cortisol levels (F(2,151) = 5.7, p = .005), with post-hoc analyses confirming a significant increase in cortisol after the MIST paradigm for all conditions. However, there was no significant interaction with condition (F(4,151) = 1.0, p = .41). No significant effect of condition was found (F(2,79) = 0.46, p = .63). Repeated measures ANOVA also confirmed a significant effect of timepoint on α-amylase levels (F(3,233) = 23.0, p < .001), but there was no significant interaction with condition (F(6,233) = 0.1, p = .71). No significant between-conditions effect was found (F(2,80) = 0.32, p = .73). Cortisol and α-amylase scores throughout the paradigm are shown in Fig. 4, Fig. 5, respectively.

### 3.4 Mindfulness associations

**Fig. 6:**
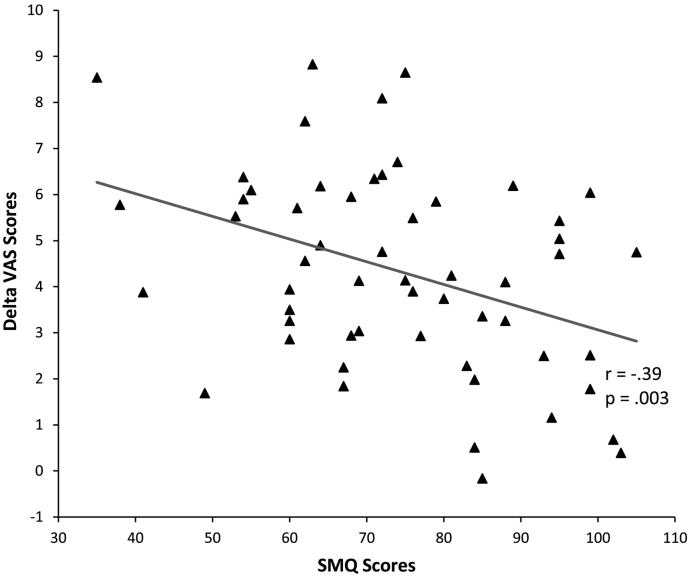
Scatterplot of Delta VAS scores and SMQ scores with correlation line.

Bivariate correlation showed that SMQ-voices scores were negatively associated with total VAS delta scores (r = −0.39, p = .003; Fig. 6). Post-hoc analyses showed that this relationship was only significant in the negative (r = −0.40, p = .036) but not in the neutral (r = 0.06, p = .77) condition. However, as shown in Table 1, independent *t*-test revealed a significant effect of voice condition on SMQ scores (t(54) = 3.9, p < .001), with significantly lower SMQ scores, indicating lower mindfulness, in the negative voice condition compared with the neutral voice condition. Further bivariate correlations showed no significant association between SMQ scores and either log_n_ AUC_g_ cortisol (r = −0.23, p = .08) or log_n_ AUC_g_ α-amylase (r = 0.03, p = .85).

## 4 Discussion

### 4.1 Findings

We had predicted that negative simulated voices would increase psychological and biological responses to stress. Our main finding indicated that, as predicted, negative voices increased the subjective stress response compared with neutral voices and ambient sounds. The effect of neutral voices and ambient sounds on subjective stress reactivity did not differ, indicating that the effect is specific to negative voices rather than voices per se.

However, this finding was not mirrored in cortisol or α-amylase levels: differences across time points indicated that the MIST affected cortisol and alpha-amylase responses, but there were no differences across conditions. A recent study demonstrated that pharmacological suppression of both HPA- and ANS-activity via dexamethasone and propranolol does not alter the subjective emotional response to psychosocial stress in healthy individuals (Ali et al., 2017), suggesting that psychological and physiological stress measures can vary independently.

We also found that more mindful appraisals of voices were associated with lower subjective stress-reactivity in the negative voice condition. This finding is in line with trials of mindfulness for clinical voice-hearers, which found evidence of reduced voice distress following therapy (Chadwick et al., 2009, Chadwick et al., 2016; López-Navarro et al., 2015; Shawyer et al., 2007). Thus, our findings lend some experimental support to the notion that mindfulness-based coping strategies may be a useful strategy to alleviate distress associated with voices.

### 4.2 Strengths and limitations

A strength of the present study is the development of simulated voices of negative and neutral content for use in research that have face validity and evoke differing levels of distress. We took great care in developing the simulated voices, consulting clinicians and voice-hearers on both the content and delivery of the voices in an iterative process. Whilst previous research has suggested simulated hallucinations are seen as realistic depictions by psychosis patients (Banks et al., 2004), to our knowledge this is the first research to show differences in affective reactions depending on voice content. Nevertheless, it is important not to assume generalization of findings from research using simulated voices to the experience of clinical voice hearers. Several aspects of AVHs, such as their interactive nature, personalised comments or auditory source location, are difficult to simulate; the simulated voices had a clear external origin due to headphone presentation, and there were ethical limits on how derogatory the content could be. Also, future research is needed to determine the impact of the simulated voices in other contexts, and not only under conditions of environmental stress.

The use of randomization to control for individual differences in prior stress exposure and stress-reactivity, and the blinding of the experimenter to condition, further add to the strength of the present study. A marked limitation of the present study is that it remains unclear as to whether the lack of biomarker findings is related to an independence of these markers from voice-content (and subjective stress levels) in actual voice-hearers as well. Alternatively, HPA- and ANS-parameters may not be sufficiently sensitive to reveal subtle differences in stress levels, which are better captured by subjective reports. A further limitation is the possibility that verbal feedback from the experimenter may have interfered with the simulated voices. Lastly, the lack of a no-stress control group means the present analysis did not investigate the effects of voices alone on stress response. However, it seems unlikely that simulated voices alone would initiate a stress response, beyond some mild negative affect in the negative voice condition.

### 4.3 Implications and future directions

In the context of the existing literature on voice content in psychosis, the present findings suggest that negative voice content may be an important factor in the subjective stress-reactivity and distress of clinical voice-hearers. Future research should investigate the predictive value of negative voice content in transition rates of at-risk populations, rather than merely the presence of voices, potentially aiding early identification of at-risk individuals and allowing for early intervention. The present subjective stress findings may have further implications for understanding the development and maintenance of psychotic disorders. According to a recent stress feedback model proposed by Howes and colleagues (Howes et al., 2016), stress exposure may exacerbate dopaminergic dysregulation, which then leads to greater levels of delusional ideation and aberrant salience, increasing need for care and distress and thus maintaining this cycle. Increased stress levels due to negative voice content may putatively contribute to dopamine dysfunction and the formation of delusional beliefs. In line with this, the effect of the present paradigm on delusional ideation, e.g., state paranoia, should be assessed in future research. Further, whilst the present study found evidence that mindful appraisal of voices is associated with attenuated subjective stress-reactivity, future research should address experimentally whether purposefully employed mindful response styles to voices also attenuate stress-reactivity, or whether mindful response styles are simply more prevalent in individuals with greater stress resilience.

## 5 Conclusions

Participants exposed to voices with negative content showed an increased subjective stress reaction to a psychosocial stressor compared with those who were exposed to voices with neutral content or an ambient sounds condition. No significant effect of voice condition on either HPA- or ANS-function were observed. The present study underscores the importance of addressing the content of voices in psychosis research and psychological therapies for voices and adds support to the emergence of mindfulness-based therapies for negative, distressing voices.

## Appendix A Supplementary data

Supplementary material Image 1
